# Schizophrenia in Thailand: prevalence and burden of disease

**DOI:** 10.1186/1478-7954-8-24

**Published:** 2010-08-17

**Authors:** Pudtan Phanthunane, Theo Vos, Harvey Whiteford, Melanie Bertram, Pichet Udomratn

**Affiliations:** 1 Setting Priorities Using Information on Cost-Effectiveness (SPICE) project, Ministry of Public Health, Nonthaburi, Thailand; 2 School of Population Health, the University of Queensland, Herston, QLD, Australia; 3 Queensland Centre for Mental Health Research, The Park, Wacol, QLD, Australia; 4 Department of Psychiatry, Faculty of Medicine, Prince of Songkla University, Songkhla, Thailand

## Abstract

**Background:**

A previous estimate of the burden of schizophrenia in Thailand relied on epidemiological estimates from elsewhere. The aim of this study is to estimate the prevalence and disease burden of schizophrenia in Thailand using local data sources that recently have become available.

**Methods:**

The prevalence of schizophrenia was estimated from a community mental health survey supplemented by a count of hospital admissions. Using data from recent meta-analyses of the risk of mortality and remission, we derived incidence and average duration using DisMod software. We used treated disability weights based on patient and clinician ratings from our own local survey of patients in contact with mental health services and applied methods from Australian Burden of Disease and cost-effectiveness studies. We applied untreated disability weights from the Global Burden of Disease (GBD) study. Uncertainty analysis was conducted using Monte Carlo simulation.

**Results:**

The prevalence of schizophrenia at ages 15-59 in the Thai population was 8.8 per 1,000 (95% CI: 7.2, 10.6) with a male-to-female ratio of 1.1-to-1. The disability weights from local data were somewhat lower than the GBD weights. The disease burden in disability-adjusted life years was similar in men (70,000; 95% CI: 64,000, 77, 000) and women (75,000; 95% CI: 69,000, 83,000). The impact of using the lower Thai disability weights on the DALY estimates was small in comparison to the uncertainty in prevalence.

**Conclusions:**

Prevalence of schizophrenia was more critical to an accurate estimate of burden of disease in Thailand than variations in disability weights.

## Background

Schizophrenia is one of the most severe and disabling mental illnesses. It is not a common disease. However, it has significant health, economic, and social consequences. In adults, the median prevalence from a systematic review was 3.3 per 1,000, ranging from 1.3 for the 10^th ^percentile to 8.2 for the 90^th ^percentile [1]. In 2001, schizophrenia ranked among the top 10 leading causes of years lived with disability (YLD) worldwide [2,3]. In Thailand in 1999, it was the eighth and ninth leading cause of YLD in men and women, respectively, responsible for 5% of disability from all causes [4]. Measured in disability-adjusted life years (DALYs), it ranked as the third-largest mental disorder after depression and anxiety disorders [4].

Apart from the significant health consequences, the health care costs of schizophrenia are also high and frequently underestimated. The World Health Organization (WHO) estimates the direct health care costs of schizophrenia in Western countries range between 1.6% and 2.6% of total health care expenditures [5]. In Taiwan, schizophrenia accounted for 1.2% of national health care expenditures [6]. To the best of our knowledge, there are no studies reporting the cost of schizophrenia in Thailand.

The burden of schizophrenia in Thailand has not been well-established. The main reason was a lack of population-based epidemiological data. The disease parameters used for the 1999 Thai Burden of Disease study, such as prevalence estimates and relative risk of death, were extrapolated from international studies [4]. Recently, more empirical data, including data from systematic reviews and local epidemiological information such as the 2003 National Mental Health Survey, have become available [7-9]. However, in the absence of incidence and disease duration data required for burden of disease estimates, modeling techniques are required to assist in estimating those missing parameters [7,10].

There has been criticism of the technical basis and value judgments used in developing the disability weights (DW) applied in burden of disease studies (such as the Global Burden of Disease). The main criticisms were: (a) that a small group of international health experts determined all DWs; and (b) that regional and cultural differences in health state valuations were ignored [11]. We carried out a study among Thai patients in contact with mental health services for schizophrenia to estimate severity and derive a local alternative of a treated disability weight in contrast with GBD assumptions.

The two objectives of the present study therefore were to (1) estimate the prevalence of schizophrenia in Thailand and (2) to estimate the burden of schizophrenia in terms of DALYs.

## Methods

### National prevalence estimates

The national prevalence of schizophrenia was estimated from a community mental health survey supplemented by a count of hospital admissions at the time of the survey. The mental health survey sample included 11,700 individuals aged between 15 and 59 years living in a residential house during the period of the survey from June to August, 2003 [9,12].

The sampling method used in the mental health survey is described in detail elsewhere http://www.dmh.go.th/journal/[9,12]. Data were collected in two steps. First, respondents were screened for mental health problems using the mental health screening questionnaire for community [12] and the Alcohol Use Disorder Identification Test (AUDIT) [13]. The former instrument contains 24 questions and was developed by Thai researchers based on their experience with psychiatric conditions and treatments of mental disorders. Subsequently, psychiatric diagnoses were assessed in those individuals screening positive using the general Mini International Neuropsychiatry Interview (MINI) [14], which was translated to Thai language [15]. Using the MINI questionnaire, people were asked a number of questions about unusual experiences; for example, "Have you ever believed that people were spying on you, or that someone was plotting against you, or trying to hurt you?" Also, they were asked if the beliefs and experiences they described were associated exclusively with times when they were feeling depressed, high, or irritable [14,15]. Data were collected during face-to-face interviews conducted by psychiatric nurses with at least two years of experience with psychiatric patients.

As the mental health survey estimated the lifetime prevalence of psychotic disorders, we adjusted the prevalence downward to reflect the prevalence of schizophrenia only, using a 0.83 ratio of schizophrenia (including schizoaffective disorder and schizophreniform disorder) to all nonaffective psychotic disorders from the study of lifetime prevalence of psychotic and bipolar disorders in a Finnish population [16]. Although cultural differences between Finland and Thailand limit the applicability of this ratio, it was the only available ratio indicating the proportion of psychotic disorders that are schizophrenia. The proportions of cases with affective psychoses including bipolar disorder and depressive psychotic disorder in this study were estimated separately in the Thai mental health survey [9]. The survey found that the prevalence of manic episode was 0.4%, hypomania was 0.5%, and mood disorder with psychotic features was 0.4%, giving an estimated prevalence of bipolar disorder of 1.3% [9]. It is toward the upper end of what would be the expected prevalence of bipolar disorder. It is therefore unlikely that we overestimated nonaffective psychosis by counting cases of affective psychosis.

Because people living in institutions such as temples and hospitals were excluded from the mental health survey, we combined the cases found in the survey with an estimate of the number of people with schizophrenia who were in a hospital during the three-month survey period. We used the 2003 data collection system of admissions to general hospitals and a separate dataset for psychiatric hospital admissions collected by the Department of Mental Health, Thailand, to estimate hospital-based prevalence. There were 1,800 patients with schizophrenia identified during the survey period in general hospitals and 4,300 in the database for psychiatric hospital admissions. We assumed no overlap between cases identified in the household mental health survey and the hospital database. Prevalent cases from each source were combined by age and sex to give an estimate of the overall prevalence of schizophrenia.

### Incidence and disease duration

We used an Incidence-Prevalence-Mortality (IPM) model, DisMod, to estimate the epidemiological parameters of schizophrenia [10]. The model assumes a causal relationship between incidence and prevalence and takes remission, cause-specific mortality, and overall mortality as competing risks into account to calculate incidence and disease duration [17,18]. If three disease parameters are defined, DisMod uses a set of mathematical equations to derive an internally consistent set of epidemiological parameters including the missing ones. In our case, these inputs were: (1) prevalence; (2) relative risk of mortality, and (3) remission rate.

**Figure 1:**
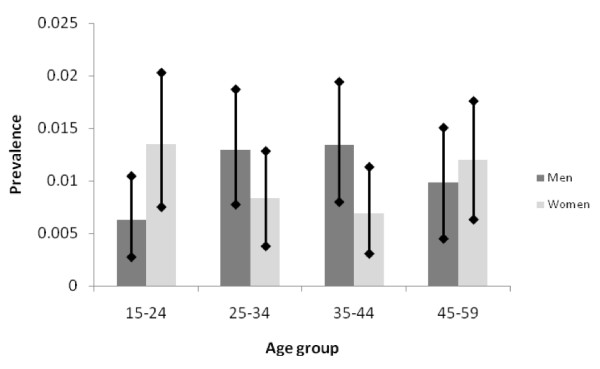
**Community-based prevalence estimates of psychotic disorders by age and sex and their 95% confidence intervals**.

We imposed an age pattern of prevalence estimated by pooling data from international prevalence studies with detailed age breakdowns identified in a recent systematic review [7] while constraining estimates to the totals estimated for ages 15-59 (Table 1). This was done for two reasons: (1) the Thai mental health survey did not report on prevalence in the elderly; and (2) age-specific estimates were based on small numbers of cases and had wide and overlapping confidence intervals (Figure 1). The relative risk of mortality in patients with schizophrenia was taken from a recent meta-analysis that reported a standardized mortality ratio for people with schizophrenia of 2.58 in both males and females [19]. A detailed age pattern for the standardized mortality ratio was available from two studies and applied so that the overall mortality ratio remained 2.58 [20,21]. As the third input parameter, we used a pooled annual remission rate of 1.37% based on 12 studies [7].

**Table 1:** The number of community-based and hospitalized people with psychotic disorders and prevalence of schizophrenia

Age	Survey participants		Surveyed cases of psychosis		Estimated community-based prevalence (percent)^a^		Hospital-based prevalence (percent)^a^	
	Male	Female	Male	Female	Male	Female	Male	Female
15-24	1,434	1,330	9	18	0.524	1.130	0.011	0.004
25-34	1,547	1,554	20	13	1.079	0.698	0.037	0.008
35-44	1,490	1,589	20	11	1.121	0.579	0.023	0.007
45-59	1,322	1,419	13	17	0.854	1.026	0.015	0.008

Total	5,793	5,889	62	59	0.900	0.848	0.022	0.007

^a ^We adjusted the number of people with psychosis to estimate schizophrenia prevalence, using the ratio of 0.83 [16].

### Disability weights

The disability weights used in GBD studies reflect the severity of nonfatal disease outcomes. Disability is defined as "any restriction or lack of ability (resulting from an impairment, any loss or abnormality of psychological or anatomical structure or function) to perform an activity in the manner or within the range considered normal for a human being" [22].

GBD studies have relied on a set of DWs that were based on the opinions of an expert group using the person trade-off method [22,23]. Placed on a disability scale of 0 to 1, where 0 represents perfect health, DWs of 0.637 for untreated schizophrenia and 0.351 for treated schizophrenia were used in GBD studies [22].

We present two different methods to capture the severity of disease from a survey we carried out among Thai people treated for schizophrenia [24]. We collected information on disease severity from both the patients' point of view using the six-dimensional EuroQoL Instrument (EQ-5D+) measuring mobility, self-care, usual activities, pain or discomfort, and anxiety/depression [25] and clinicians' ratings on the Brief Psychiatric Rating Scale Expanded version (BPRS-E) [26]. For the first method, we used a multiplicative regression model developed for Australian Burden of Disease studies [27]. This regression model determined the relationship between 241 health states with EQ-5D+ descriptors and the disability weights from the Dutch Burden of Disease study [25,27]. Given the weights from that regression, we mapped the EQ-5D+ scores as reported by each patient in our survey [24] into those disability weights [25,27]. We then computed the average disability weights by age and sex. The second method is based on that developed for the Assessing Cost-Effectiveness Mental Health project in Australia to translate severity measured as a change in BPRS-E score into a DW [28,29]. They implemented a sliding scale between the highest and lowest values of the Dutch disability weights for schizophrenia into a health status measure of each respondent from the Australian mental health survey to calculate an average disability weight [25,28,29]. Here, we assumed the highest and lowest BPRS-E scores in our study sample correspond to the highest and lowest values of the Dutch disability weights for schizophrenia [25], and all other BPRS-E values are spread linearly across this range of DW values.

In the 1999 Thai Burden of Disease study, it was estimated that 63% of cases were undergoing treatment and 37% were untreated, with an average GBD disability weight of 0.452 [30]. We retained this assumption in our current study because the mental health survey did not collect information able to elicit this proportion. We used the GBD DW for untreated patients, while the three different measures of DW (Thai survey methods based on EQ5D+ and BPRS as well as GBD weights) were used for treated patients to get the possible range of overall disease burden.

### Burden of Disease

People with schizophrenia normally die from indirect causes, such as an increased risk of suicide and lifestyle risk factors leading to physical disorders (e.g., respiratory and cardiovascular diseases). Therefore, there are no deaths coded directly to schizophrenia and hence no years of life lost (YLL) due to premature mortality but only YLD contributing to DALY estimates of schizophrenia [19,31]. There are two ways to compute YLD: incident YLD and prevalent YLD [32,33]. A discount rate of 3% was applied to incident YLD, but no GBD age-weighting function was applied [3].

We calculated the uncertainty around YLD estimates using the uncertainty surrounding the epidemiological parameters and the DWs as input parameters. First, we used the DisMod program, implementing a method called "parametric bootstrapping" http://www.epigear.com to generate multiple samples of modeled incidence, prevalence, and disease duration. As input variables, we defined a binomial distribution for survey prevalence and remission (Table 1). A triangular distribution was specified for the standardized mortality ratio. We ran 500 iterations, generating a range of results within which we believed the true value would fall. Next, we used Ersatz http://www.epigear.com to combine the uncertainty around incidence and duration with that of the disability weights to determine uncertainty ranges around YLD.

## Results

The majority of people with schizophrenia were in the community (98%) (Table 1). The survey prevalence of schizophrenia in the Thai population aged between 15-59 years in 2003 was 8.8 per 1,000 (95% CI: 7.2, 10.6) with a male-to- female ratio of 1.1-to-1.

The modeled incidence rate was 0.3 per 1,000, with a peak at ages 15-24 in both males and females (Table 2). Prevalence peaked at ages between 30 and 44 in males and at ages between 40 and 54 in females. The disease duration in females was longer than in males across all age groups due to greater life expectancy. The average duration of schizophrenia was 30 and 34 years in men and women, respectively.

**Table 2:** Modeled incidence, prevalence, and disease duration by age groups and gender in 2005

Age group (years)	Incidence per 1,000		Prevalence per 1,000		Duration (years)	
	male	female	male	female	male	female
15-24	0.8	0.6	7.1	4.4	31	38
25-34	0.5	0.5	11.9	8.6	28	34
35-44	0.2	0.4	12.5	11	24	29
45-54	0	0.1	11.2	12	21	25
55-64	0	0	9.4	10.8	15	18
65+	0	0	6.7	8.4	10	11

Overall	0.3	0.28	8.06	7.30	30	34
Male-to-female sex ratio		1.11		1.10		

The patient- and clinician-rated DWs were similar, with very little variation by age or sex (Table 3). It was therefore considered reasonable to use a single DW for all ages and both sexes in our estimates. The local DWs were somewhat lower than the GBD-treated DW (Table 3).

**Table 3:** Disability weights estimated based on different approaches

Age	Untreated DW	Treated DW		
	GBD	GBD	EQ-5D+	BPRS
15-29	0.627	0.351	0.317	0.305

30-44	0.627	0.351	0.298	0.316

45-59	0.645	0.351	0.319	0.323

Total	0.627	0.351	0.308 (0.284 to 0.333)^a^	0.316 (0.305 to 0.327)

Males	0.627	0.351	0.314 (0.282 to 0.345)	0.316 (0.300 to 0.332)

Females	0.627	0.351	0.299 (0.259 to 0.340)	0.316 (0.300 to 0.333)

The 95% confidence intervals were estimated based on nonparametric bootstrapping methods using Stata 10.0 (StataCorp, Texas, USA).

**Figure 2:**
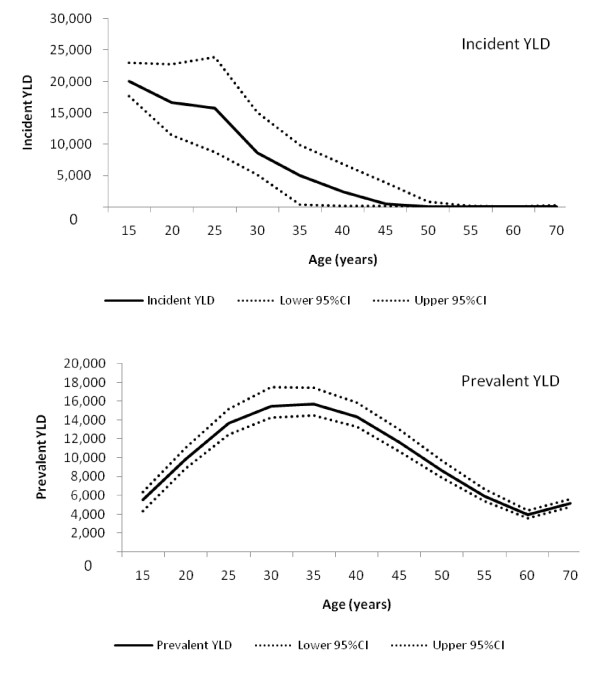
**Incident and prevalent YLD in males and their 95% confidence intervals**.

**Figure 3:**
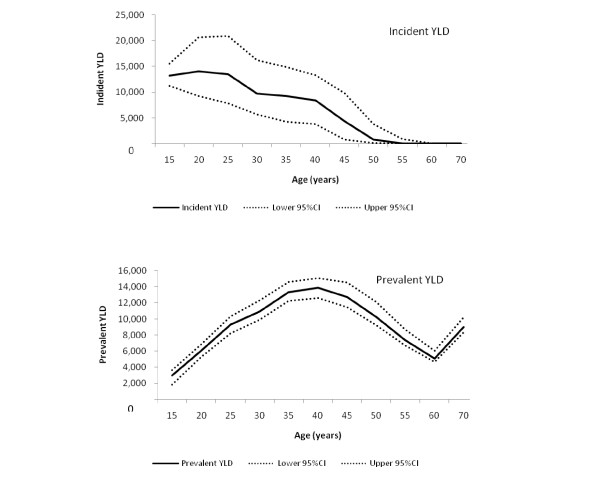
**Incident and prevalent YLD in females and their 95% confidence intervals**.

Figures 2 and 3 show the age pattern of incident and prevalent YLD with their 95% confidence intervals. Incident YLD was 75,000 (95% CI: 69,000, 83,000) in women and 70,000 (95% CI: 64,000, 77,000) in men, with the somewhat lower prevalence in women being compensated by longer duration estimates. In 2005, prevalent YLD in men (110,000; 95% CI: 100,000, 120,000) was marginally higher than in women (100,000; 95% CI: 96,000, 110,000), reflecting the higher prevalence in men than in women.

**Figure 4:**
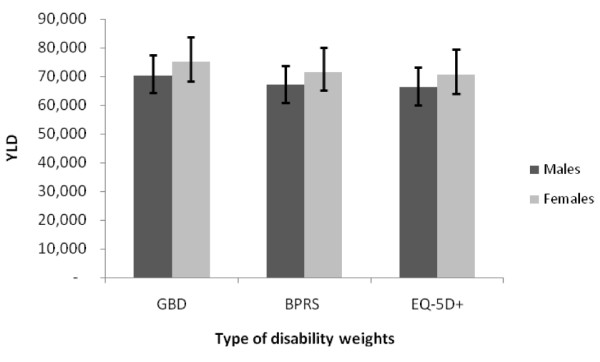
**Incident YLD across YLD using three disability weight estimates**.

Using DWs derived from local data or GBD assumptions had no significant impact on YLD estimates in men or women (Figure 4).

## Discussion

This is the first estimation of the burden of disease due to schizophrenia in Thailand based on Thai prevalence and severity data. It is also the first study undertaking uncertainty analyses to determine the influence of uncertain epidemiological variables on the burden of disease from schizophrenia.

Our prevalence estimate of 8.8 per 1,000 falls in the highest decile of global prevalence figures reported in a recent meta-analysis [1]. It is also higher than the prevalence ranges from 2.7 to 8.3 per 1,000 reported by two other reviews [34,35]. It is, however, difficult to compare our prevalence estimate with these other estimates for a number of reasons: (1) differences in strategy for data collection (community household surveys, register or case note (institutionalized) information, or a combination of them); (2) differences in screening methods and diagnostic criteria (ICD-10 and DSM-IV or an earlier version of either classification system); (3) differences in defined population (our estimate pertains to 15- to 59-year-olds only); and (4) differences in field work planning (quality of training programs for interviewers). We found no sex differential in prevalence. This is consistent with the review study by Saha [1] and a recent population-based survey in Finland [16]. Another mental health survey was conducted in 2008. However, the final results are not yet available. When data become available, they can be used to update these estimates.

The YLD of schizophrenia were higher in Thailand than in the GBD studies in 2004 due to a higher Thai prevalence estimate [36]. Using the data provided by WHO, the average YLD of schizophrenia per capita across the world and among Southeast Asian countries was estimated to be 0.002 and 0.001, respectively [37], while this study found YLD per capita of 0.004 in Thailand. The GBD study used the mean prevalence of 0.004. The disability burden in 2005 is lower than that estimated in 1999 in total, with a higher burden in females but lower in males [4]. However, the difference in the data used limits comparison between the two studies. In 1999, a prevalence estimate of 7 per 1,000 in men and 8 per 1,000 in women was based on expert judgment. Less important are small differences in estimates of remission and risk of mortality.

No other study has reported on DWs using individual data on EQ-5D+ in the mental health area. The disability weight derived from the BPRS was used in an Australian economic evaluation study to calculate the health gain from interventions [28,29], but no previous study has incorporated this approach into burden of disease. Despite local data giving smaller DWs than those used in GBD studies, replacing universal weights with the local weights does not significantly alter YLD. Partly this is due to the fact that the relatively large uncertainty around epidemiological parameters leads to an overlap in estimates; the main explanation is that our local DWs for treated schizophrenia were similar to the disability weight for treated psychosis in GBD. While the DALY approach has been criticized for using expert panels to identify disease severity [11], our findings suggest that experts, clinicians, and patients do not rate the severity of treated schizophrenia very differently. The uncertainty around our prevalence estimates was more important. Three additional factors were not included in our quantification of uncertainty. First, the mental health survey may have underestimated prevalence due to the stigma of schizophrenia. Second, using a ratio of schizophrenia to total nonaffective psychotic disorders from a Finnish study introduces further uncertainty. Third, we assumed that people diagnosed with schizophrenia were in either a hospital or in the community, and that none of them was in both places through the three-month survey period. However, since data gathered by the Department of Mental Health suggested that the average length of inpatient stay at psychiatric hospitals in Thailand was 71 days in 2003 [38], this assumption would be reasonable.

## Conclusions

Sound epidemiological data, including incidence, prevalence, disability weight, and duration, are key factors in estimating burden of disease. However, the results suggest that an accurate estimate of the prevalence of schizophrenia is more critical than the variation in disability weights estimated from different perspectives.

## Competing interests

The authors declare that they have no competing interests.

## Authors' contributions

PP, TV, and HW conceptualized the research. PP, TV, and MB conducted the data analysis. PU provided clinical and epidemiological expertise in the Thai context. PP wrote the first draft of the manuscript. All authors have been involved in reviewing the manuscript and have given final approval of this manuscript version.
